# Normal and reference values for cardiovascular magnetic resonance-based pulse wave velocity in the middle-aged general population

**DOI:** 10.1186/s12968-021-00739-y

**Published:** 2021-04-19

**Authors:** Max J. van Hout, Ilona A. Dekkers, Jos J. Westenberg, Martin J. Schalij, Ralph L. Widya, Renée de Mutsert, Frits R. Rosendaal, Albert de Roos, J. Wouter Jukema, Arthur J. Scholte, Hildo J. Lamb

**Affiliations:** 1 Department of Cardiology, Leiden University Medical Center, Albinusdreef 2, 2333 ZA Leiden, The Netherlands; 2 Department of Radiology, Leiden University Medical Center, Albinusdreef 2, 2333 ZA Leiden, The Netherlands; 3 Department of Epidemiology, Leiden University Medical Center, Albinusdreef 2, 2333 ZA Leiden, The Netherlands

**Keywords:** Pulse Wave Velocity, Magnetic Resonance Imaging, Arterial stiffness, Cardiovascular disease, Epidemiology

## Abstract

**Background:**

Aortic stiffness, assessed through pulse wave velocity (PWV), is an independent predictor for cardiovascular disease risk. However, the scarce availability of normal and reference values for cardiovascular magnetic resonance imaging (CMR) based PWV is limiting clinical implementation. The aim of this study was to determine normal and reference values for CMR assessed PWV in the general population.

**Methods:**

From the 2,484 participants of the Netherlands Epidemiology of Obesity (NEO) study that have available CMR-PWV data, 1,394 participants free from cardiovasculard disease, smokers or treatment for diabetes, hypertension or dyslipidaemia were selected (45–65 years, 51% female). Participants were divided into sex, age and blood pressure (BP) subgroups. Normal values were specified for participants with a BP < 130/80 mmHg and reference values for elevated BP subgroups (≥ 130/80 and < 140/90 mmHg; and ≥ 140/90 mmHg). Differences between groups were tested with independent samples t-test or ANOVA. Due to an oversampling of obese individuals in this study, PWV values are based on a weighted analysis making them representative of the general population.

**Results:**

Normal mean PWV was 6.0 m/s [95% CI 5.8–6.1]. PWV increased with advancing age and BP categories (both p < 0.001). There was no difference between sex in normal PWV, however in the BP > 140/90 mmHg women had a higher PWV (p = 0.005). The interpercentile ranges were smaller for participants < 55 years old compared to participants ≥ 55 years, indicating an increasing variability of PWV with age. PWV upper limits were particularly elevated in participants ≥ 55 years old in the high blood pressure subgroups.

**Conclusion:**

This study provides normal and reference values for CMR-assessed PWV per sex, age and blood pressure category in the general population.

**Supplementary Information:**

The online version contains supplementary material available at 10.1186/s12968-021-00739-y.

## Background

Arterial stiffness is a marker of vascular disease and is becoming increasingly important in cardiovascular risk assessment [1, 2]. The pulse wave velocity (PWV) has arisen as the standard for the assessment of arterial stiffness. There are many technical variants of PWV assessment, where cardiovascular magnetic resonance (CMR) assessed PWV and applanation tonometry based carotid-femoral PWV (cf-PWV) are well-established techniques and independent predictors of cardiovascular disease (CVD) [1, 2]. These two techniques yield different PWV values, most likely due to the inability of cf-PWV to accurately assess aortic length [3] cf-PWV has some other important limitations as it can be difficult to obtain a signal in patients with obesity and local aortic PWV assessment is not possible [3]. CMR-PWV is a promising technique for clinical use, however normal and reference values for CMR assessed PWV are scarce, currently limiting the clinical application [4–8].

In addition, age and sex specific normal and reference values for PWV are important for personalized CVD risk assessment, where over the last decades increasing evidence and awareness has emerged towards the difference in cardiovascular aging between men and women [9]. Only a few, relatively small, studies have provided age and sex specific normal ranges for CMR-based PWV [4–8]. Although PWV may be a predictor of CVD on top of traditional risk factors as age and systolic blood pressure (BP), it is well known that PWV is also strongly dependent on age and BP [10, 11]. For this reason it is imperative to define normal PWV values for patients with normal BP and reference values for patients with elevated blood pressures in a population free of CVD, smokers or known hypertension, dyslipidaemia and diabetes, as has been done for cf-PWV [12].

The aim of this study was to define both normal values (normal BP) and reference values (high BP) stratified per sex and age category for CMR-based PWV in a population free of CVD.

## Methods

### Study population and study design NEO study

The present study is a cross-sectional analysis of the baseline measurements in the Netherlands Epidemiology of Obesity (NEO) study, a population-based, prospective cohort study in 6,671 men and women between 45 and 65 years [13]. Men and women living in the greater area of Leiden (the Netherlands) were invited to participate in the study if they were aged between 45 and 65 years and had a self-reported body mass index (BMI) of ≥ 27 kg/m^2^. In addition, all inhabitants from one municipality (Leiderdorp) were invited to participate irrespective of their BMI, allowing for a reference distribution of BMI (n = 1,671). Participants completed a general questionnaire to report demographic, lifestyle and clinical information. At the baseline visit, all participants underwent an extensive physical examination including anthropometry and blood sampling. Participants with potential contraindications for CMR (i.e. metallic devices, claustrophobia, or a body circumference of 1.70 m) were excluded for additional imaging. Approximately 35% of the participants without potential CMR contraindications were randomly selected for abdominal MRI including PWV. For the present analysis, we included only data of individuals who underwent CMR PWV. Participants with overt CVD, smokers or participants who are treated for diabetes, hypertension or dyslipidaemia were excluded from the sample used, because these patients are known to have a significantly higher PWV [12]. The Medical Ethical Committee of the Leiden University Medical Center (LUMC) approved the design of the study and all participants provided written informed consent.

### Data collection NEO study

The participants were asked to bring all medication they were using to the study visit. Medication used in the month preceding the study visit was recorded. On the questionnaire, participants reported ethnicity by self-identification, tobacco smoking, highest level of education, and alcohol consumption using a food frequency questionnaire (in grams/day). Brachial BP was measured in a seated position on the right arm using a validated automatic oscillometric device (OMRON, Model M10-IT, Omron Health Care Inc, Kyoto, Japan). BP was measured three times with 5 min rest between consecutive measurements. The mean systolic and diastolic BP were calculated [13]. Blood samples were drawn from the antecubital vein after 5 min rest of the participant during the baseline visit. Fasting plasma glucose and serum insulin concentrations, haemoglobin A1c (HbA1c), high sensitivity C-reactive protein (hsCRP), serum total cholesterol, low-density lipoprotein (LDL) and high-density lipoprotein (HDL) cholesterol were determined in the central clinical chemistry laboratory of the LUMC by using standard methods [13].

### Cardiovascular magnetic resonance imaging

**Fig. 1:**
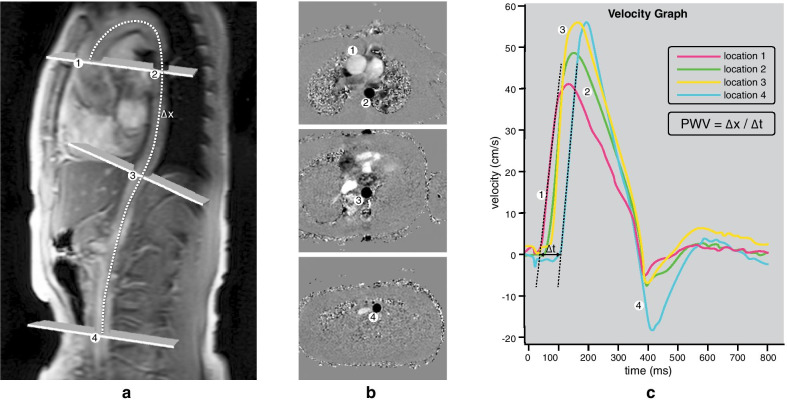
Cardiovascular magnetic resonance (CMR) aortic pulse wave velocity (PWV) assessment: **a **Through-plane flow measurements at the level of the pulmonary trunk cutting across both the ascending and the proximal descending aorta, just below the diaphragm perpendicular to the descending aorta and just above the bifurcation of the abdominal aorta. **b** Corresponding velocity-encoded images. **c** PWV is subsequently calculated from the distance along the aortic centerline between measurement locations (Δx) and the foot-to-foot transit time from the resulting velocity waveforms (Δt)

CMR was performed on a 1.5 T scanner (Philips Medical Systems, Best, the Netherlands) [13]. Aortic PWV (expressed in meters per second) was imaged by using a previously described protocol, illustrated in Fig. 1 [14]. In summary, a scout view of the entire aorta was obtained. A retrospectively electrocardiogram (ECG)-gated gradient-echo sequence with velocity encoding was performed during free breathing to assess flow using the following imaging parameters: field-of-view 300 mm, rectangular field-of-view percentage 90%, repetition time 4.8 ms, echo time 2.8 ms, flip angle 20°, acquired voxel size 2.34 × 2.34 × 8.00 mm, reconstructed voxel size 1.17 × 1.17 × 8.00 mm, temporal resolution 9.6 ms (defined by 2 times the repetition time) and on average 171 reconstructed phases. The velocity encoding was set to a maximum of 200 cm/sec. These through-plane flow measurements were performed at the level of the pulmonary trunk perpendicular to the ascending aorta, just below the diaphragm perpendicular to the descending aorta and just above the bifurcation of the abdominal aorta. To establish a high temporal resolution, a maximum number of phases was reconstructed. Maximum velocity–time curves from each sampling sited provided the arrival time of the systolic pressure wave. The foot of the systolic wave front was detected automatically using in-house developed software, by assessing the intersection point of the horizontal diastolic flow and the upslope of the systolic wave front, modelled by a linear regression along the upslope from the flow values between 20 and 80% of the range. The aortic path length between the measurement sites was measured using MASS software (Medis, Leiden, The Netherlands) The aortic path length was divided by the transit time between the arrival of the systolic wave front at these sites to calculate the PWV. Accurate assessment of PWV over shorter distances is more difficult due to the shorter transit time requiring a higher temporal resolution, therefore in this manuscript we focus on PWV of the entire aorta. PWV of the entire aorta was assessed as a weighted mean of the PWV sampled at these 4 measurement points, as adding sampling points along the aorta increases the accuracy of PWV measurement [15]. The weighted mean was calculated by averaging the PWV of the 4 segments in proportion to their segment length.

### Statistical analysis

In the NEO study individuals with a BMI of 27 kg/m^2^ or higher were oversampled. First, inhabitants of Leiden and its surrounding area between 45 and 65 years of age and with a self-reported BMI of 27 kg m^-2^ or higher were invited to participate in the NEO study. In addition, all inhabitants between 45 and 65 years living in one municipality, Leiderdorp, were asked to participate irrespective of their BMI. This resulted in an additional sample of 1671 participants with a BMI distribution that was similar to the BMI distribution of the general Dutch population [16]. If inference is made on the general population, the overrepresentation of overweight and obese participants in the NEO study may introduce bias because of the skewed BMI distribution in the NEO population. Weighting towards the BMI distribution of the general population solves this problem [17]. Using the reference BMI distribution of the Leiderdorp population, weight factors for the NEO population were calculated, resulting in a higher weight factor for participants with a lower BMI. Use of sampling weights yields results that apply to a population-based study without oversampling of individuals with a high BMI [18]. Consequently, the results apply to a population-based study without oversampling of individuals with a BMI ≥ 27 kg/m^2^. Additional file 1: Figure S1 shows that the use of the weight factors results in a BMI distribution that is similar to that of the general population. This method has been used extensively in NEO study publications, among others to address oversampling in generating reference values [19].

We performed a complete case analysis. Normal values were presented as mean with 95% confidence interval (CI) and median with 10th, 25th, 75th and 90th percentiles in NEO participants stratified by sex (men/ women) and age (45.0 to 49.9, 50.0 to 54.9, 55.0 to 59.9, 60.0 to 64.9 years old) category. Furthermore, normal values were provided for the normal (< 130/80 mmHg) blood pressure subgroup, and reference values were provided for high BP subgroups (stage 1: ≥ 130/80 and < 140/90 mmHg; stage 2: ≥ 140/90 mmHg) according to the American College of Cardiology (ACC)/American Heart Association (AHA) guidelines.[20] Differences between groups were tested with either a two-tailed independent samples t-test or ANOVA. Analyses were performed using STATA (Stata Corporation, College Station, Texas, USA) and results are presented according to the STROBE guidelines [21].

## Results

**Fig. 2:**
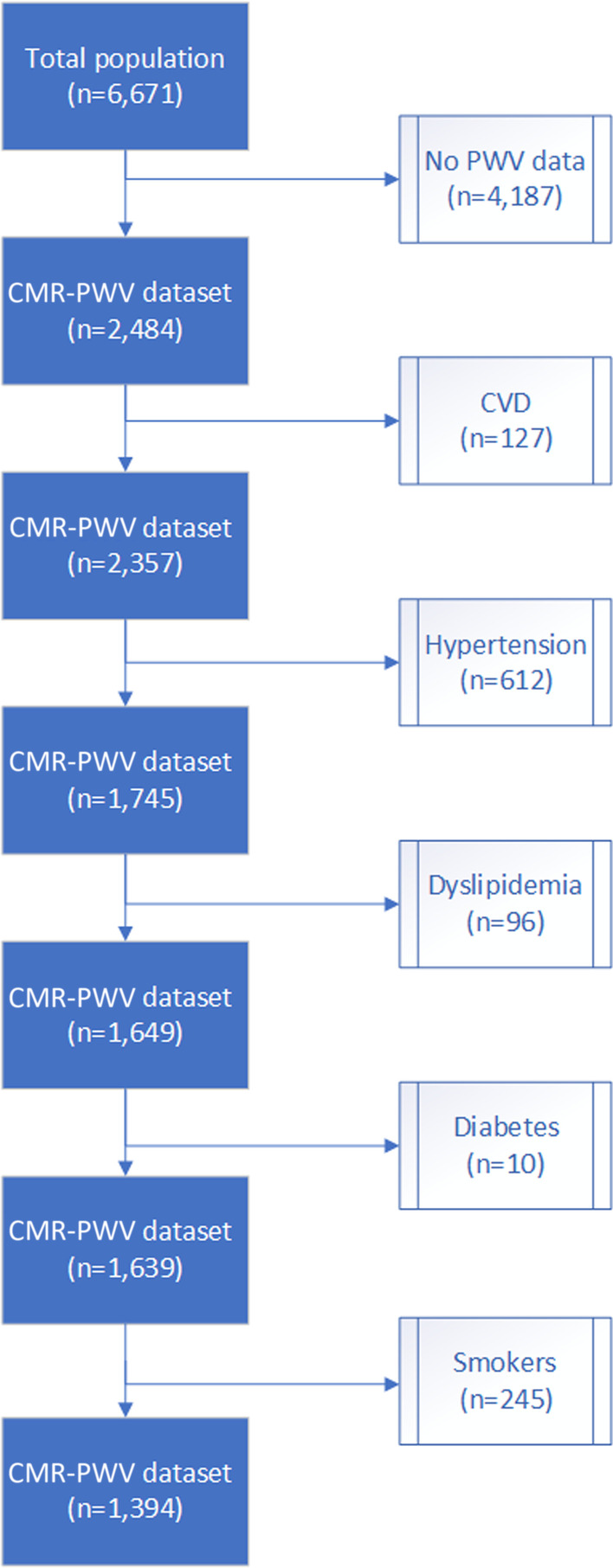
Flowchart illustrating population sample selection

In total 6,671 individuals participated in the NEO study of whom a random subset of 2,484 participants without contraindications for CMR underwent CMR-PWV assessment. After exclusion of patients with cardiovascular disease (CVD), smokers and treatment for hypertension, diabetes or dyslipidaemia, the final study population consisted of 1,394 participants (flowchart is shown in Fig. 2), with a mean (SD) age of 55 [6] years, of whom 51% is female. Physical and demographic parameters of the final study population stratified by sex are summarized in Table 1, characteristics of excluded participants are shown in Additional file 1: Table S1.

**Table 1:** Characteristics of the study population grouped by sex

	**Men** n = 684 (49%)	**Women** n = 710 (51%)	**Total** n = 1,394
Demographic/anthropometric			
Age (years)	55.1 ± 6.4	55.0 ± 5.6	55.0 ± 6.0
Height (m)	1.81 ± 0.07	1.67 ± 0.06	1.73 ± 0.10
Weight (kg)	86.1 ± 13.0	69.5 ± 11.5	76.8 ± 14.7
BMI (kg/m^2^)	26.1 ± 3.4	24.9 ± 3.8	25.4 ± 3.7
Heart rate (beats/min)	66.9 ± 10.7	69.1 ± 9.4	68.1 ± 10.1
Alcohol intake (g/day)	18.4 ± 19.4	9.5 ± 10.4	13.4 ± 15.4
Education level (% high)	53.8	46.5	49.7
Ethnicity (% white)	95.4	94.5	94.9
Blood pressure			
Systolic (mmHg)	133.6 ± 15.8	126.2 ± 17.3	129.5 ± 17.2
Diastolic (mmHg)	84.6 ± 10.8	81.6 ± 10.0	82.9 ± 10.5
Biomarkers			
Total cholesterol (mmol/L)	5.7 ± 1.0	5.8 ± 1.0	5.8 ± 1.0
HDL (mmol/L)	1.4 ± 0.3	1.8 ± 0.4	1.6 ± 0.4
LDL (mmol/L)	3.7 ± 0.9	3.6 ± 1.0	3.6 ± 0.9
Fasting glucose (mmol/l)	5.5 ± 1.1	5.2 ± 0.6	5.3 ± 0.8
eGFR (ml/min/1,73 m^2^)	86.0 ± 13.4	83.1 ± 13.8	84.4 ± 13.8
hsCRP (mg/L)	1.7 ± 2.9	1.8 ± 2.5	1.8 ± 2.6

Data are shown as % or mean ± SD. *BMI* body mass index, *eGFR* estimated glomerular filtration rate, *HDL* high-density lipoprotein, *hsCRP* high sensitivity C-reactive protein, *LDL* low-density lipoprotein

In this study population, in which participants treated for hypertension have been excluded, we provide normal values (n = 397) for the normal BP subgroup, and reference values for stage 1 (n = 474) and stage 2 hypertension (n = 523) BP subgroups.

### Normal values for PWV

**Fig. 3:**
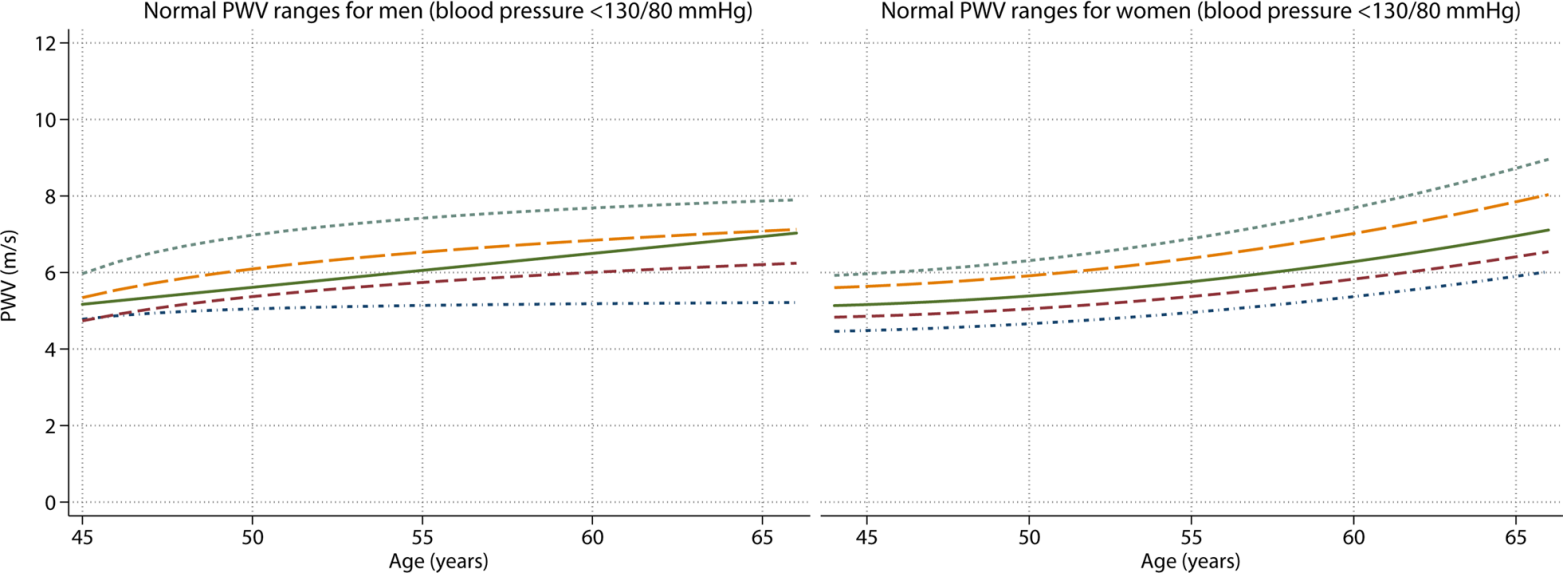
Normal PWV ranges for age range 45–65 years in men and women. From bottom line to top: 10th, 25th, median, 75th and 90th percentile

Normal values for PWV stratified by age are presented in Table 2 and normal values additionally stratified by sex are shown in Table 3. PWV increased with advancing age, where PWV increased on average 0.9 [0.7–1.0] m/s every 10 years (p < 0.001). The mean normal PWV for the age category 45 to 50 years old was 5.4 [95% CI: 5.3–5.6] m/s whereas it was 6.8 [95% CI: 6.5–7.0] m/s in the age category of 60 to 65 years (Table 2). There was no difference in PWV between men and women (overall mean of 6.0 [95% CI 5.8–6.3] m/s for men and 6.0 [95% CI 5.8–6.1] m/s for women, p = 0.57). Up to the age of 60 years, men had a slightly higher mean PWV than women, however in the age group of 60 to 65 years, women have a slightly higher PWV than men (Table 3). This rising trend in female PWV as compared to male PWV is also illustrated in Fig. 3, where the normal age and sex specific PWV values are shown (with 10th, 25th, median, 75th and 90th percentile lines). For comparison, an overview of previously published studies defining CMR-based PWV values in the age range 45–65 years is provided in Table 4.

**Table 2:** Normal values for CMR-PWV in m/s stratified per age category (n = 397)

Age (years)	Mean [95% CI]	Median [10–90^th^ pc]
PWV (m/s)		
45 to < 50	5.4 [5.3–5.6]	5.4 [4.6–6.5]
50 to < 55	5.8 [5.6–5.9]	5.6 [5.0–6.5]
55 to < 60	6.1 [5.8–6.5]	6.0 [5.0–7.1]
60 to < 65	6.8 [6.5–7.0]	6.8 [5.7–7.9]

*PWV* pulse wave velocity

**Table 3:** Normal and reference values for CMR-PWV stratified per sex, age and blood pressure category

Age (years)	Normal values (n = 397) BP < 130/80 mmHg		Stage 1 HTN* (n = 474) BP ≥ 130/80, < 140/90 mmHg		Stage 2 HTN* (n = 523) BP ≥ 140/90 mmHg	
	Mean [95% CI]	Median [10-90th pc]	Mean [95% CI]	Median [10-90th pc]	Mean [95% CI]	Median [10-90th pc]
PWV (m/s)						
Men						
45 to < 50	5.6 [5.3–6.0]	5.5 [4.9–6.8]	5.6 [5.5–5.8]	5.4 [5.1–6.2]	6.0 [5.8–6.3]	6.0 [5.2–6.9]
50 to < 55	5.8 [5.6–6.1]	5.8 [5.1–6.5]	6.0 [5.7–6.3]	5.9 [5.1–7.2]	6.2 [6.0–6.5]	6.0 [5.5–7.3]
55 to < 60	6.2 [5.8–6.7]	6.1 [5.3–7.8]	6.7 [6.0–7.3]	6.3 [5.6–7.8]	7.0 [6.6–7.4]	6.8 [5.6–8.8]
60 to < 65	6.6 [6.2–7.1]	6.8 [5.4–8.0]	7.0 [6.6–7.4]	6.7 [5.9–9.3]	7.5 [7.1–7.9]	7.3 [5.8–9.5]
Women						
45 to < 50	5.3 [5.1–5.5]	5.2 [4.6–6.1]	5.6 [5.4–5.8]	5.5 [4.9–6.5]	6.2 [6.0–6.4]	6.2 [5.6–7.1]
50 to < 55	5.7 [5.5–5.9]	5.6 [5.0–6.5]	6.3 [6.0–6.7]	6.1 [5.4–8.0]	6.6 [6.2–7.0]	6.5 [5.7–7.7]
55 to < 60	6.1 [5.7–6.5]	5.8 [5.0–7.0]	6.9 [6.5–7.3]	6.7 [5.6–8.3]	7.4 [7.0–7.8]	7.2 [6.1–8.7]
60 to < 65	6.8 [6.5–7.1]	6.8 [5.7–7.9]	7.3 [6.9–7.8]	7.0 [5.9–9.8]	8.5 [7.8–9.3]	7.9 [6.4–12.4]

*BP* blood pressure, *HTN* hypertension, *PWV* pulse wave velocity. *Categorized according to the ACC/AHA guidelines

**Table 4:** Overview of CMR population studies defining PWV values in the age range 45–65 years

Study	Population size (n)	CMR PWV technique	Age range (years)	CVD risk profile	Median PWV range (m/s) Men	Median PWV range (m/s) Women	PWV (m/s) between 45 and 65
2011. Westenberg et al.	25	gradient-echo multi-slice through-plane	18–63	Healthy volunteers	4.5–6.7	4.5–6.7	5.5–6.9
2013 Kim et al.	124	gradient-echo multi-slice through-plane	20–79	Healthy volunteers	4.2–6.5	4.2–6.5	4.9–6.5
2015. Nethononda et al.	777	gradient-echo multi-slice through-plane	21–85	Population (incl. HTN, diabetics and smokers)	4.4–13.0	4.3–11.9	6.5–9.6
2018. Harloff et al.	126	4D-flow	20–80	Population (incl. HTN, diabetics and smokers)	4.4–7.8	4.6–8.5	5.2–8.1

*CVD* cardiovascular disease, *HTN* hypertension, *PWV* pulse wave velocity

### Reference values for PWV

**Fig. 4:**
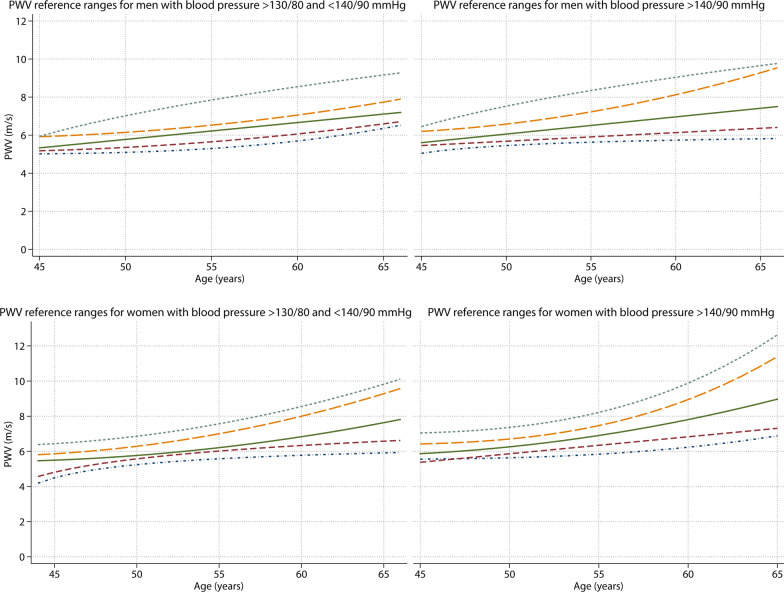
Distribution of CMR-PWV reference ranges per blood pressure category (stage 1 and 2 hypertension*) for age range 45–65 years in the general population. From bottom line to top: 10th, 25th, median, 75th and 90th percentile. *categorized according to the ACC/AHA guidelines

Reference values stratified by age and sex are presented in Table 3. In the high BP (stage 1 and 2) reference populations, PWV increased slightly faster with advancing age than the normal values did; where PWV increased on average 1.0 [0.8–1.3] m/s every 10 years for stage 1 (p < 0.001) and 1.3 [0.9–1.7] m/s every 10 years in stage 2 hypertension subgroups (p < 0.001). Mean PWV was higher in participants with a high BP (6.8 [95% CI 6.6–6.9] m/s) as compared to normal BP (6.0 [95% CI 5.8–6.1] m/s, p < 0.001). Mean and median PWV values were sequentially higher in increasing BP subgroups in both men and women (Table 3 and Fig. 4).

In contrast to the normal population, in the reference populations women consistently had a slightly higher PWV than men (Table 3). Similar to the normal population there was no overall difference in PWV between sexes for stage 1 hypertension (mean of 6.3 [95% CI 6.1–6.6] m/s for men and 6.5 [95% CI 6.3–6.8] m/s for women, p = 0.24). However, in the stage 2 hypertension subgroup women had a higher PWV than men (mean of 6.8 [95% CI 6.6–7.0] m/s for men and 7.4 [95% CI 7.1–7.8] m/s for women, p = 0.005). Women showed an increasingly rising trend for PWV values with age, particularly in the 90^th^ percentile lines, as was also observed in the normal values for women. The difference between men and women is consequently only observed in the age subgroup of 60 to 65 years old (PWV for men 7.5 [95% CI 7.1–7.9] m/s and for women 8.5 [95% CI 7.8–9.3] m/s, p = 0.02). These results are illustrated in Fig. 4, where the age and sex specific reference PWV values are shown (with 10th, 25th, median, 75th and 90th percentile lines).

Normal and reference values per aortic segment can be found in the supplemental tables (Additional file 1: Tables S2-S7). Normal PWV values per aortic segment in the weighted analysis versus the reference (Leiderdorp) population (general population not selected based on BMI of ≥ 27 kg/m^2^) are shown in Additional file 1: Table S8.

## Discussion

In this population-based study of 1,394 participants, normal and reference ranges for CMR-based PWV were established in the general population free of CVD and treatment for hypertension, diabetes or hypercholesterolemia. This is the largest study to date to provide age and sex specific normal values (for normal BP < 130/80 mmHg) and reference values (for stage 1 (≥ 130/80; < 140/90 mmHg) and stage 2 hypertension (≥ 140/90 mmHg)) for CMR-PWV. Defining normal values and reference values is essential for the implementation of CMR-PWV in clinical care.

### Normal and reference values for PWV

CMR assessment of aortic pathology is gaining clinical relevance, since it provides the opportunity for simultaneous assessment of aortic dimensions together with aortic stiffness, distensibility, blood flow and wall shear stress. Normal and reference values for cf-PWV based on applanation tonometry have previously been established by the Reference Values for Arterial Stiffness Collaboration Group, who determined age, sex and BP specific normal and reference values in a pooled study population consisting of 11,092 participants [12]. This group reported higher normal PWV values than we did, for example, mean and median PWV was more than 2 m/s higher in the 50 to 60 years old age group. Given the low risk profile of both populations, the difference in normal PWV values is most likely explained by the use of different PWV techniques (applanation tonometry versus CMR), illustrating the need for CMR-PWV specific normal and reference values. The ability to accurately assess aortic length is crucial for accurate PWV assessment, whereas cf-PWV requires the use of a correction factor of 0.8 to adjust for the overestimation of aortic length [22, 23]. Since aortic length is known to increase with age, this correction factor is known to perform worse with aging which is an important limitation of cf-PWV that does not apply to CMR-PWV [24].

Beside different PWV techniques such as cf-PWV, there are also differences in CMR-PWV scanning techniques, for example, techniques based on 2D through-plane or in-plane phase-contrast or 4D flow, in which 4D flow has the advantage of providing local PWV and additional parameters as wall shear stress at the cost of a higher temporal resolution [25, 26]. Most of these techniques are in good agreement with invasive intra-aortic pressure measurements [25]. This is also the case for the multi-slice through-plane phase-contrast PWV used in the current study, which is the most widely available and most user friendly technique [14, 25].

Previous studies that determined normal and reference values for CMR-PWV were limited by their sample size and/or defined in a specific age range (e.g. children and adolescents) [6]. We observed similar normal PWV values as compared to a small study performed in an Asian population (n = 124), which had a similar CVD risk profile and used the same CMR-PWV technique [5]. A small investigation with 25 healthy subjects using a similar CMR-PWV technique showed comparable normal PWV values as compared to our results [7]. One of the larger previous investigations (n = 777), also using gradient-echo multi-slice through-plane PWV, found higher PWV reference values as compared to our study, which can be explained by the fact that they included patients treated for diabetes, hypercholesterolemia and hypertension, subgroups known to have increased PWV values [4]. A recent study provided reference values for 4D-flow CMR-PWV (n = 126), which found consistently higher PWV values for men [8]. Also they found slightly higher PWV values as compared to our population, particularly for men, which may also be attributed to the inclusion of participants with diabetes, hypertension and hypercholesterolemia. An overview of these studies can be found in Table 4.

In our population there were no differences between men and women in CMR-PWV values, except in the highest age and BP subgroup. Sex differences in PWV normal and reference values remain a topic of debate as previous studies found conflicting results regarding sex differences [4–6, 8, 12]. These varying results can partially be explained by the used sample sizes in combination with the included age distribution, where cardiovascular sex differences are likely to become more pronounced after menopause [27]. The median and 90th percentile PWV for men and women showed a diverging trend with advancing age, with a stronger increase in women (Fig. 3). Women are known to have a lower CVD risk before menopause but this risk increases after menopause around the age of 55 as is also observed in our CMR-PWV normal and reference values [27]. The hormonal changes during menopause can lead to changes in body fat composition, cholesterol metabolism, impaired endothelial function and inflammatory processes which effect vascular structure and accelerates vascular dysfunction leading to increased CVD risk [28].

Consistent with our current knowledge of vascular stiffness etiology, normal CMR-PWV values increased with age, with an average increase of 0.9 m/s every 10 years between the age of 45 to 65. Age affects aortic stiffness through complex inflammatory and oxidative stress pathways disrupting endothelial and smooth muscle cell function and extracellular matrix composition [29]. The variability of PWV also increased with age, as the 90^th^ percentile showed a stronger increase compared to the 10^th^ percentile, demonstrating the variability of vascular aging in the general population.

The close relation between PWV and BP has been previously captured in mathematical functions, as PWV is known to increase with BP [10]. Even though participants with treatment for hypertension were excluded, there was still a large variation in PWV values between normal and high BP subgroups. The variability of PWV values was relatively small in the normal BP population, however this increased substantially in the high BP groups. The ACC/AHA BP guideline used in this study is stricter than the European Society of Cardiology (ESC) guideline, which defines hypertension as a BP above 140/90 mmHg [20, 30]. Therefore, depending on which definition is used, the BP category ≥ 130/80 and < 140/90 mmHg can also be regarded as (high) normal. The ESC guideline currently uses a cut-off for cf-PWV of 10 m/s as abnormal, however a single cut-off for PWV does not capture the variability in CVD risk between different age groups and BP ranges [30]. Given how important age, sex and BP related changes are in affecting aortic stiffness, specific reference values are imperative for identification of high risk patients using CMR-PWV.

### Limitations

Although this large population study provides necessary CMR-PWV normal and reference values, there are also some limitations to consider. Because there was a oversampling of obese participants in the NEO study, adjustments were performed by weighting individuals towards the BMI distribution of participants in the Leiderdorp subpopulation [31]. As a result, the normal and reference values do apply to the general population between 45 and 65 years. This study provides normal and reference values for the age range 45 to 65 years, an important age group for CVD risk stratification, however there is no information available for other age groups outside these ranges in this study. Although we used the weighting factor to achieve a population that is representative of the general population and have excluded participants with known CVD, smokers and participants who were being treated for diabetes, hypertension or dyslipidemia, this is a population study. Therefore, PWV can variate more as compared to a selected healthy population due to variations in among others body height, weight, other medication use, chronic infections, exercise and dietary habits. The normal values in this study apply to the average population with a normal BP without overt CVD. The NEO population exists for 95% of Caucasian participants, so the use of these normal and reference values in areas with different ethnic compositions should be done with caution, as arterial stiffness assessed with PWV has shown to differ between ethnic groups.[32] Furthermore, it should be recognized that BP was assessed at the initial visit (3 consecutive measurement with 5 min rest in between), where measurement over multiple visits or assessment of 24-h ambulatory BP would have been preferable.

## Conclusion

This study provides normal and reference values for CMR-assessed PWV per sex, age and BP category in the middle-aged general population free of CVD. These values provide incremental information in CVD risk assessment on top of traditional risk factors, such as age and BP, making them essential for the clinical application of CMR in the assessment of cardiovascular pathology.

## Supplementary Information

**Additional file 1.** Additional figures and tables.

## Data Availability

The datasets generated and/or analysed during the current study are not publicly available due to the privacy of the participants of the NEO study and legal reasons, but will be made available to qualified researchers by the NEO Executive Board upon reasonable request, which can be contacted via https://www.lumc.nl/org/neostudie/contact/.

## Abbreviations

ACC/AHA: American College of Cardiology/American Heart Association
BMI: Body mass index
BP: Blood pressure
cf-PWV: Carotid-femoral pulse wave velocity
CMR: Cardiovascular magnetic resonance
CVD: Cardiovascular disease
ECG: Electrocardiogram
ESC: European Society of Cardiology
HbA1c: Hemoglobin A1c
HDL: High density lipoprotein
hsCRP: High sensitivity C-reactive protein
HTN: Hypertension
LDL: Low density lipoprotein
NEO: Netherlands Epidemiology of Obesity
PWV: Pulse wave velocity

## References

[1] Ben-Shlomo Y, Spears M, Boustred C, May M, Anderson SG, Benjamin EJ (2014). Aortic pulse wave velocity improves cardiovascular event prediction: an individual participant meta-analysis of prospective observational data from 17,635 subjects. J Am Coll Cardiol.

[2] Maroules CD, Khera A, Ayers C, Goel A, Peshock RM, Abbara S (2014). Cardiovascular outcome associations among cardiovascular magnetic resonance measures of arterial stiffness: the Dallas heart study. J Cardiovasc Magn Reson.

[3] Pereira T, Correia C, Cardoso J (2015). Novel methods for pulse wave velocity measurement. J Med Biol Eng.

[4] Nethononda RM, Lewandowski AJ, Stewart R, Kylinterias I, Whitworth P, Francis J (2015). Gender specific patterns of age-related decline in aortic stiffness: a cardiovascular magnetic resonance study including normal ranges. J Cardiovasc Magn Reson.

[5] Kim EK, Chang SA, Jang SY, Kim Y, Kim SM, Oh JK (2013). Assessment of regional aortic stiffness with cardiac magnetic resonance imaging in a healthy Asian population. Int J Cardiovasc Imaging.

[6] Voges I, Jerosch-Herold M, Hedderich J, Pardun E, Hart C, Gabbert DD (2012). Normal values of aortic dimensions, distensibility, and pulse wave velocity in children and young adults: a cross-sectional study. J Cardiovasc Magn Reson.

[7] Westenberg JJ, Scholte AJ, Vaskova Z, van der Geest RJ, Groenink M, Labadie G (2011). Age-related and regional changes of aortic stiffness in the Marfan syndrome: assessment with velocity-encoded MRI. J Magn Reson Imag.

[8] Harloff A, Mirzaee H, Lodemann T, Hagenlocher P, Wehrum T, Stuplich J (2018). Determination of aortic stiffness using 4D flow cardiovascular magnetic resonance - a population-based study. J Cardiovasc Magn Reson.

[9] Suzuki H, Kondo K (2013). Pulse Wave Velocity in Postmenopausal Women. Pulse (Basel, Switzerland).

[10] Ma Y, Choi J, Hourlier-Fargette A, Xue Y, Chung HU, Lee JY (2018). Relation between blood pressure and pulse wave velocity for human arteries. Proc Natl Acad Sci USA.

[11] Mitchell GF, Hwang SJ, Vasan RS, Larson MG, Pencina MJ, Hamburg NM (2010). Arterial stiffness and cardiovascular events: the Framingham Heart Study. Circulation.

[12] Determinants of pulse wave velocity in healthy people and in the presence of cardiovascular risk factors: 'establishing normal and reference values'. Eur Heart J. 2010;31(19):2338–50. 10.1093/eurheartj/ehq165 PMC2948201 20530030

[13] de Mutsert R, den Heijer M, Rabelink TJ, Smit JW, Romijn JA, Jukema JW (2013). The Netherlands Epidemiology of Obesity (NEO) study: study design and data collection. Eur J Epidemiol.

[14] Grotenhuis HB, Westenberg JJ, Steendijk P, van der Geest RJ, Ottenkamp J, Bax JJ (2009). Validation and reproducibility of aortic pulse wave velocity as assessed with velocity-encoded MRI. J Magn Resonan Imag.

[15] Kroner ES, van der Geest RJ, Scholte AJ, Kroft LJ, van den Boogaard PJ, Hendriksen D (2012). Evaluation of sampling density on the accuracy of aortic pulse wave velocity from velocity-encoded MRI in patients with Marfan syndrome. J Magn Resonan Imag.

[16] Bakel AM, Zantinge EM. Hoeveel mensen hebben overgewicht of ondergewicht?(How many people are underweight or overweight?). Natl Kompas Volksgezond. 2010.

[17] Korn EL, Graubard BI (1991). Epidemiologic studies utilizing surveys: accounting for the sampling design. Am J Public Health.

[18] Lumley T (2004). Analysis of complex survey samples. J Stat Softw.

[19] Kroon FPB, Ramiro S, Royston P, Le Cessie S, Rosendaal FR, Kloppenburg M (2017). Reference curves for the Australian/Canadian Hand Osteoarthritis Index in the middle-aged Dutch population. Rheumatology (Oxford).

[20] Whelton PK, Carey RM, Aronow WS, Casey DE, Jr., Collins KJ, Dennison Himmelfarb C, et al. 2017 ACC/AHA/AAPA/ABC/ACPM/AGS/APhA/ASH/ASPC/NMA/PCNA Guideline for the Prevention, Detection, Evaluation, and Management of High Blood Pressure in Adults: Executive Summary: A Report of the American College of Cardiology/American Heart Association Task Force on Clinical Practice Guidelines. Hypertension (Dallas, Tex : 1979). 2018;71(6):1269–324. 10.1161/HYP.0000000000000066 29133354

[21] von Elm E, Altman DG, Egger M, Pocock SJ, Gotzsche PC, Vandenbroucke JP (2008). The Strengthening the Reporting of Observational Studies in Epidemiology (STROBE) statement: guidelines for reporting observational studies. J Clin Epidemiol.

[22] Huybrechts SA, Devos DG, Vermeersch SJ, Mahieu D, Achten E, de Backer TL (2011). Carotid to femoral pulse wave velocity: a comparison of real travelled aortic path lengths determined by MRI and superficial measurements. J Hypertens.

[23] Weber T, Ammer M, Rammer M, Adji A, O'Rourke MF, Wassertheurer S (2009). Noninvasive determination of carotid-femoral pulse wave velocity depends critically on assessment of travel distance: a comparison with invasive measurement. J Hypertens.

[24] Van Bortel LM, Laurent S, Boutouyrie P, Chowienczyk P, Cruickshank JK, De Backer T (2012). Expert consensus document on the measurement of aortic stiffness in daily practice using carotid-femoral pulse wave velocity. J Hypertens.

[25] Wentland AL, Grist TM, Wieben O (2014). Review of MRI-based measurements of pulse wave velocity: a biomarker of arterial stiffness. Cardiovasc Diagn Ther.

[26] Westenberg JJ, de Roos A, Grotenhuis HB, Steendijk P, Hendriksen D, van den Boogaard PJ (2010). Improved aortic pulse wave velocity assessment from multislice two-directional in-plane velocity-encoded magnetic resonance imaging. J Magn Resonan Imag.

[27] Kannel WB, Hjortland MC, McNamara PM, Gordon T (1976). Menopause and risk of cardiovascular disease: the Framingham study. Ann Intern Med.

[28] Agarwala A, Michos ED, Samad Z, Ballantyne CM, Virani SS (2020). The use of sex-specific factors in the assessment of women's cardiovascular risk. Circulation.

[29] Donato AJ, Machin DR, Lesniewski LA (2018). Mechanisms of dysfunction in the aging vasculature and role in age-related disease. Circ Res.

[30] Williams B, Mancia G, Spiering W, Agabiti Rosei E, Azizi M, Burnier M (2018). 2018 ESC/ESH Guidelines for the management of arterial hypertension. Eur Heart J.

[31] Dekkers IA, de Mutsert R, de Vries APJ, Rosendaal FR, Cannegieter SC, Jukema JW (2018). Determinants of impaired renal and vascular function are associated with elevated levels of procoagulant factors in the general population. J Thromb Haem.

[32] Santos PC, Alvim Rde O, Ferreira NE, de Sa CR, Krieger JE, Mill JG (2011). Ethnicity and arterial stiffness in Brazil. Am J Hypertens.

