# Supplementary material for: Normal and reference values for cardiovascular magnetic resonance-based pulse wave velocity in the middle-aged general population
Source: J Cardiovasc Magn Reson. 2021 Apr 19;23:46. doi: 10.1186/s12968-021-00739-y (PMC8054386; doi:10.1186/s12968-021-00739-y)
Supplement: Supplementary file 1 — Additional file 1. Additional figures and tables. [file 12968_2021_739_MOESM1_ESM.docx]

**Figure S1.** Distribution of BMI in the Leiden population (Blue line) and in the total NEO population (red) before weighting (left) and after weighting (right).

**Table S1.** Baseline characteristics of participants included and excluded in this study (reason for exclusion is provided in the flow chart (figure 2)).

|  | | **Included (n=1,394)** | | **Excluded (n=5,277)** | |  |
| --- | --- | --- | --- | --- | --- | --- |
|  | | **Men**  n = 684 (49%) | **Women**  n = 710 (51%) | **Men**  n = 2,472  (47%) | **Women**  n = 2,805  (53%) | **Total**  n = 6,671 |
| **Demographic/anthropometric** | |  |  |  |  |  |
|  | Age (years) | 55.1 ± 6.4 | 55.0 ± 5.6 | 56.4 ± 6.2 | 55.6 ± 5.8 | 55.7 ± 6.0 |
|  | Height (m) | 1.81 ± 0.07 | 1.67 ± 0.06 | 1.81 ± 0.07 | 1.67 ± 0.06 | 1.73 ± 0.10 |
|  | Weight (kg) | 86.1 ± 13.0 | 69.5 ± 11.5 | 88.6 ± 14.5 | 73.3 ± 14.2 | 79.2 ± 15.9 |
|  | BMI (kg/m^2^) | 26.1 ± 3.4 | 24.9 ± 3.8 | 27.1 ± 4.0 | 26.2 ± 4.9 | 26.3 ± 4.4 |
|  | Heart rate (beats/min) | 66.9 ± 10.7 | 69.1 ± 9.4 | 65.7 ± 11.1 | 68.7 ± 9.8 | 67.6 ± 10.4 |
|  | Alcohol intake (g/day) | 18.4 ± 19.4 | 9.5 ± 10.4 | 21.0 ± 20.1 | 10.6 ± 11.8 | 14.7 ± 16.3 |
|  | Education level (% high) | 53.8 | 46.5 | 46.0 | 43.6 | 45.9 |
|  | Ethnicity (% white) | 95.4 | 94.5 | 95.0 | 94.7 | 94.9 |
|  | |  |  |  |  |  |
| **Blood pressure** | |  |  |  |  |  |
|  | Systolic (mmHg) | 133.6 ± 15.8 | 126.2 ± 17.3 | 134.6 ± 16.2 | 126.9 ± 16.6 | 130.1 ±17.0 |
|  | Diastolic (mmHg) | 84.6 ± 10.8 | 81.6 ± 10.0 | 84.8 ± 10.4 | 81.9 ± 9.9 | 83.1 ± 10.3 |
|  | |  |  |  |  |  |
| **Biomarkers** | |  |  |  |  |  |
|  | Total cholesterol (mmol/L) | 5.7 ± 1.0 | 5.8 ± 1.0 | 5.5 ± 1.1 | 5.8 ± 1.0 | 5.7 ± 1.1 |
|  | HDL (mmol/L) | 1.4 ± 0.3 | 1.8 ± 0.4 | 1.3 ± 0.4 | 1.7 ± 0.4 | 1.6 ± 0.5 |
|  | LDL (mmol/L) | 3.7 ± 0.9 | 3.6 ± 1.0 | 3.5 ± 1.0 | 3.5 ± 0.9 | 3.5 ± 1.0 |
|  | Fasting glucose (mmol/l) | 5.5 ± 1.1 | 5.2 ± 0.6 | 5.7 ± 1.2 | 5.4 ± 0.8 | 5.5 ± 1.0 |
|  | eGFR (ml/min/1,73 m^2^) | 86.0 ± 13.4 | 83.1 ± 13.8 | 87.6 ± 15.2 | 83.6 ± 14.0 | 85.1 ± 14.4 |
|  | hsCRP (mg/L) | 1.7 ± 2.9 | 1.8 ± 2.5 | 2.0 ± 2.9 | 2.4 ± 3.1 | 2.1 ± 3.0 |
|  |  |  |  |  |  |  |

*Data are shown as % or mean ± SD. Abbreviations: BMI; body mass index, eGFR; estimated glomerular filtration rate, HDL; high-density lipoprotein, hsCRP; high sensitivity C-reactive protein, LDL; low-density lipoprotein.*

**Table S2.** Normal values for aortic arch CMR-PWV in m/s stratified per age category (n=397)

|  | | **PWV** (m/s) |  |
| --- | --- | --- | --- |
| **Age** (years) | | **Mean** [95% CI] | **Median** [10-90^th^ pc] |
|  | |  |  |
|  | *45 to <50* | 5.8 [5.4 - 6.2] | 5.7 [4.1 - 7.5] |
|  | *50 to <55* | 6.1 [5.6 - 6.6] | 5.6 [4.5 - 7.9] |
|  | *55 to <60* | 6.5 [6.0 - 7.0] | 6.3 [4.7 - 8.5] |
|  | *60 to <65* | 6.5 [6.0 - 7.1] | 6.0 [4.5 - 8.7] |
|  | |  |  |

*Mean ± SD segment transit distance 110.9 ± 14.6 mm and time 17.7 ± 7.2 ms.*

**Table S3.** Normal and reference values for aortic arch PWV stratified by sex, age and blood pressure category.

| **PWV arch** (m/s) | | | | | | |
| --- | --- | --- | --- | --- | --- | --- |
| **Age** (years) | **Normal values** (n=397)  *BP <130/80 mmHg* | | **Stage 1 HTN*** (n=474)  *BP ≥130/80, <140/90 mmHg* | | **Stage 2 HTN*** (n=523)  *BP ≥140/90 mmHg* | |
|  | Mean  [95% CI] | Median  [10-90^th^ pc] | Mean  [95% CI] | Median  [10-90^th^ pc] | Mean  [95% CI] | Median  [10-90^th^ pc] |
| ***Men*** |  |  |  |  |  |  |
| *45 to <50* | 5.8 [5.4 - 6.2] | 5.9 [4.7 - 6.9] | 5.6 [5.2 - 5.9] | 5.3 [4.4 - 7.1] | 6.0 [5.6 - 6.3] | 5.8 [4.7 - 7.1] |
| *50 to <55* | 6.2 [5.1 - 7.4] | 5.5 [4.0 - 8.1] | 6.4 [6.0 - 6.8] | 6.2 [5.0 - 7.3] | 6.3 [5.8 - 6.8] | 5.9 [4.7 - 8.3] |
| *55 to <60* | 6.1 [5.6 - 6.6] | 6.3 [4.7 - 7.4] | 7.0 [5.9 - 8.1] | 6.3 [4.8 - 12.6] | 6.9 [6.3 - 7.4] | 6.8 [4.2 - 8.7] |
| *60 to <65* | 6.7 [6.1 - 7.3] | 6.1 [5.1 - 7.9] | 6.6 [6.1 - 7.1] | 6.3 [5.2 - 8.1] | 7.6 [6.8 - 8.5] | 6.9 [5.3 - 10.5] |
| ***Women*** |  |  |  |  |  |  |
| *45 to <50* | 5.8 [5.2 - 6.3] | 5.1 [4.0 - 7.5] | 6.2 [5.3 - 7.0] | 5.5 [4.5 - 7.6] | 6.4 [6.0 - 6.9] | 6.1 [5.2 - 7.3] |
| *50 to <55* | 6.0 [5.5 - 6.5] | 5.8 [4.5 - 7.9] | 5.9 [5.4 - 6.3] | 5.6 [4.2 - 7.8] | 6.4 [5.8 - 7.0] | 6.3 [4.2 - 8.3] |
| *55 to <60* | 6.7 [6.0 - 7.3] | 6.3 [4.7 - 8.9] | 7.2 [6.1 - 8.3] | 6.3 [5.1 - 10.3] | 6.9 [6.3 - 7.6] | 6.9 [4.5 - 8.9] |
| *60 to <65* | 6.5 [5.7 - 7.2] | 6.0 [4.4 - 9.2] | 7.0 [6.3 - 7.7] | 6.7 [4.7 - 10.0] | 7.8 [7.2 - 8.4] | 7.6 [5.8 - 10.0] |
|  |  |  |  |  |  |  |

**Table S4.** Normal values for thoracic descending aortic CMR-PWV in m/s stratified per age category (n=397)

|  | | **PWV** (m/s) |  |
| --- | --- | --- | --- |
| **Age** (years) | | **Mean** [95% CI] | **Median** [10-90^th^ pc] |
|  | |  |  |
|  | *45 to <50* | 6.2 [5.6 - 6.9] | 5.7 [4.2 - 8.4] |
|  | *50 to <55* | 6.3 [5.7 - 7.0] | 6.0 [4.2 - 7.5] |
|  | *55 to <60* | 6.8 [6.3 - 7.3] | 6.5 [4.9 - 9.1] |
|  | *60 to <65* | 7.8 [7.0 - 8.6] | 7.5 [5.2 - 10.0] |
|  | |  |  |

*Mean ± SD segment transit distance 133.4 ± 19.2 mm and time 20.4 ± 9.0 ms.*

**Table S5.** Normal and reference values for thoracic descending aortic PWV stratified by sex, age and blood pressure category.

| **PWV thoracic descending** (m/s) | | | | | | |
| --- | --- | --- | --- | --- | --- | --- |
| **Age** (years) | **Normal values** (n=397)  *BP <130/80 mmHg* | | **Stage 1 HTN*** (n=474)  *BP ≥130/80, <140/90 mmHg* | | **Stage 2 HTN*** (n=523)  *BP ≥140/90 mmHg* | |
|  | Mean  [95% CI] | Median  [10-90^th^ pc] | Mean  [95% CI] | Median  [10-90^th^ pc] | Mean  [95% CI] | Median  [10-90^th^ pc] |
| ***Men*** |  |  |  |  |  |  |
| *45 to <50* | 5.6 [5.1 - 6.1] | 5.7 [4.2 - 7.2] | 6.8 [6.1 - 7.5] | 6.5 [4.8 - 7.9] | 7.0 [6.3 - 7.8] | 6.4 [5.2 - 9.6] |
| *50 to <55* | 6.9 [5.5 - 8.4] | 6.2 [4.8 - 11.1] | 6.3 [5.9 - 6.7] | 6.1 [5.0 - 7.9] | 7.3 [6.7 - 7.9] | 7.0 [4.8 - 9.5] |
| *55 to <60* | 7.4 [6.3 - 8.4] | 6.9 [4.3 - 9.8] | 7.0 [5.9 - 8.1] | 6.6 [5.0 - 11.0] | 8.6 [7.7 - 9.4] | 8.2 [5.9 - 11.6] |
| *60 to <65* | 7.3 [6.6 - 8.1] | 7.5 [4.7 - 9.1] | 8.3 [7.0 - 9.6] | 7.0 [5.5 - 15.7] | 8.4 [7.7 - 9.0] | 7.9 [6.0 - 11.0] |
| ***Women*** |  |  |  |  |  |  |
| *45 to <50* | 6.5 [5.6 - 7.4] | 5.5 [4.3 - 9.4] | 5.8 [5.4 - 6.2] | 5.7 [4.2 - 7.2] | 6.2 [5.7 - 6.7] | 5.8 [4.9 - 7.6] |
| *50 to <55* | 6.0 [5.5 - 6.4] | 6.0 [4.1 - 7.1] | 7.7 [6.4 - 9.0] | 6.7 [4.9 - 11.3] | 8.4 [6.6 - 10.2] | 7.1 [6.1 - 15.9] |
| *55 to <60* | 6.6 [6.1 - 7.1] | 6.3 [4.9 - 8.6] | 7.5 [6.7 - 8.4] | 6.9 [5.5 - 9.8] | 9.4 [7.9 - 10.9] | 8.4 [6.3 - 14.8] |
| *60 to <65* | 8.0 [6.9 - 9.1] | 6.9 [5.2 - 13.4] | 9.1 [7.4 - 10.9] | 8.3 [4.9 - 15.0] | 10.7 [9.3 - 12.2] | 10.4 [7.2 - 17.0] |
|  |  |  |  |  |  |  |

**Table S6.** Normal values for abdominal descending aortic CMR-PWV in m/s stratified per age category (n=397)

|  | | **PWV** (m/s) |  |
| --- | --- | --- | --- |
| **Age** (years) | | **Mean** [95% CI] | **Median** [10-90^th^ pc] |
|  | |  |  |
|  | *45 to <50* | 5.2 [4.9 - 5.6] | 5.0 [4.0 - 7.0] |
|  | *50 to <55* | 5.6 [5.3 - 5.8] | 5.3 [4.3 - 6.9] |
|  | *55 to <60* | 5.6 [5.2 - 6.0] | 5.4 [4.0 - 6.9] |
|  | *60 to <65* | 7.2 [6.4 - 7.9] | 6.6 [4.7 - 10.4] |
|  | |  |  |

*Mean ± SD segment transit distance 145.1 ± 24.0 mm and time 25.0 ± 12.9 ms.*

**Table S7.** Normal and reference values for abdominal descending aortic CMR-PWV stratified by sex, age and blood pressure category.

| **PWV abdominal descending** (m/s) | | | | | | |
| --- | --- | --- | --- | --- | --- | --- |
| **Age** (years) | **Normal values** (n=397)  *BP <130/80 mmHg* | | **Stage 1 HTN*** (n=474)  *BP ≥130/80, <140/90 mmHg* | | **Stage 2 HTN*** (n=523)  *BP ≥140/90 mmHg* | |
|  | Mean  [95% CI] | Median  [10-90^th^ pc] | Mean  [95% CI] | Median  [10-90^th^ pc] | Mean  [95% CI] | Median  [10-90^th^ pc] |
| ***Men*** |  |  |  |  |  |  |
| *45 to <50* | 5.9 [5.1 - 6.8] | 5.3 [4.2 - 9.0] | 5.1 [4.8 - 5.4] | 4.8 [4.2 - 6.2] | 5.9 [5.4 - 6.4] | 5.3 [4.6 - 7.8] |
| *50 to <55* | 5.7 [5.2 - 6.2] | 5.5 [4.7 - 6.9] | 5.8 [5.1 - 6.5] | 5.1 [4.3 - 8.4] | 6.3 [5.7 - 6.8] | 5.9 [4.7 - 8.6] |
| *55 to <60* | 6.1 [5.5 - 6.7] | 6.1 [4.5 - 6.9] | 7.6 [5.3 - 9.8] | 6.3 [5.2 - 10.9] | 6.4 [5.8 - 7.1] | 6.0 [4.7 - 9.1] |
| *60 to <65* | 6.5 [5.8 - 7.2] | 6.6 [4.7 - 9.2] | 7.2 [6.1 - 8.4] | 6.2 [4.7 - 11.8] | 7.4 [6.6 - 8.1] | 7.3 [4.5 - 10.5] |
| ***Women*** |  |  |  |  |  |  |
| *45 to <50* | 5.0 [4.7 - 5.3] | 4.8 [4.0 - 6.0] | 6.1 [5.1 - 7.2] | 5.6 [4.3 - 8.1] | 6.5 [6.0 - 7.0] | 6.3 [5.2 - 8.1] |
| *50 to <55* | 5.5 [5.1 - 5.8] | 5.2 [4.2 - 6.9] | 6.3 [5.4 - 7.2] | 5.8 [4.5 - 8.7] | 6.2 [5.7 - 6.7] | 5.9 [5.1 - 8.3] |
| *55 to <60* | 5.3 [4.9 - 5.8] | 5.2 [4.0 - 6.7] | 6.6 [5.9 - 7.3] | 6.4 [4.7 - 8.6] | 6.7 [6.3 - 7.2] | 7.0 [5.3 - 8.1] |
| *60 to <65* | 7.4 [6.4 - 8.5] | 6.6 [4.7 - 10.8] | 7.7 [6.9 - 8.5] | 6.6 [5.6 - 11.7] | 8.7 [6.7 - 10.7] | 6.9 [5.0 - 13.8] |
|  |  |  |  |  |  |  |

**Table S8:** Normal CMR-PWV values (BP <130/80 mmHg) in m/s stratified per age category in the weighted analysis versus the reference (Leiderdorp) population (general population not selected based on BMI of ≥27 kg/m^2^) per aortic segment.

|  | **PWV arch** (m/s) | | **PWV thoracic descending** (m/s) | | **PWV abdominal descending** (m/s) | |
| --- | --- | --- | --- | --- | --- | --- |
| **Age** (years) | Mean [95% CI] | | Mean [95% CI] | | Mean [95% CI] | |
|  | Weighted population (n=397) | Ref. Leiderdorp population  (n=142) | Weighted population  (n=397) | Ref. Leiderdorp population  (n=142) | Weighted population  (n=397) | Ref. Leiderdorp population  (n=142) |
|  |  |  |  |  |  |  |
| *45 to <50* | 5.8 [5.4 - 6.2] | 5.6 [5.2 - 6.1] | 6.2 [5.6 - 6.9] | 6.2 [5.5 – 7.0] | 5.2 [4.9 - 5.6] | 5.2 [4.8 - 5.6] |
| *50 to <55* | 6.1 [5.6 - 6.6] | 5.9 [5.3 - 6.5] | 6.3 [5.7 - 7.0] | 6.3 [5.7 - 6.9] | 5.6 [5.3 - 5.8] | 5.5 [5.2 - 5.8] |
| *55 to <60* | 6.5 [6.0 - 7.0] | 6.4 [5.8 - 7.0] | 6.8 [6.3 - 7.3] | 7.0 [6.0 - 7.9] | 5.6 [5.2 - 6.0] | 5.6 [5.1 - 6.1] |
| *60 to <65* | 6.5 [6.0 - 7.1] | 6.5 [5.7 - 7.3] | 7.8 [7.0 - 8.6] | 7.7 [6.8 - 8.6] | 7.2 [6.4 - 7.9] | 7.1 [6.3 - 7.9] |
|  |  |  |  |  |  |  |
